# Characterization of Collagen from Sakhalin Taimen Skin as Useful Biomass

**DOI:** 10.17113/ftb.58.04.20.6734

**Published:** 2020-12

**Authors:** Takeshi Nagai, Masataka Saito, Yasuhiro Tanoue, Norihisa Kai, Nobutaka Suzuki

**Affiliations:** 1 Graduate School of Agricultural Sciences, Yamagata University, Tsuruoka, 9978555 Yamagata, Japan; 2 The United Graduate School of Agricultural Sciences, Iwate University, Morioka, 0208550 Iwate, Japan; 3 Graduate School, Prince of Songkla University, 90112 Songkhla, Thailand; 4 Kagawa Nutrition University, Sakado, 3500288 Saitama, Japan; 5 Department of Food Science and Technology, National Fisheries University,; Shimonoseki, 7596595 Yamaguchi, Japan; 6 Department of Integrated Science and Technology, Oita University, Oita, 8701192 Oita,; Japan; 7 Nagoya Research Institute, Toyoake, 4701131 Aichi, Japan

**Keywords:** Sakhalin taimen skin, useful biomass, collagen, succinylation, improvement of functional property

## Abstract

**Research background:**

Animal collagen has been widely utilized in foods, cosmetics and biomedical fields. The non-edible portion, such as fish skin and bones, are obtained during cooking. Most of them are currently discarded as wastes, although the nutritional value of the skin and bones is high. The non-edible portion needs to be reused in order to reduce environmental impact, as it is one of the sources of environmental pollution.

**Experimental approach:**

Collagen was prepared by cold acetone treatment from Sakhalin taimen skin as a waste produced during cooking. Next, the colour, SDS-polyacrylamide gel electrophoresis, ultraviolet absorption, subunit composition, amino acid composition, denaturation temperature and attenuated total reflectance-Fourier transform infrared spectroscopy analyses were conducted to explore the properties of the collagen. Lastly, we attempted to improve the functional properties of the collagen for future applications using chemical modification technique (succinylation).

**Results and conclusions:**

Cold acetone treatment easily removed the fats and pigments from the skin. The odourless and pure white collagen was obtained with high yield. The α3 chain did not exist in the collagen. Sakhalin taimen skin collagen had rich α-helix and low β-sheet structures. Succinylation caused the secondary structural changes of the collagen molecule. Moreover, it made it possible not only to increase the viscosity of the collagen solution but also to improve the solubility of the collagen under the physiological conditions around pH=6.

**Novelty and scientific contribution:**

This finding is the first report on the absence of the α3 chain from salmonid fish skin collagens. The succinylated collagen from Sakhalin taimen skins as useful biomass has potential to utilize in foods, cosmetics and related industries.

## INTRODUCTION

Sakhalin taimen (*Hucho perryi*) belongs to the order Salmoniformes and is a member of salmon family. It is known as one of the largest, least understood, and most ancient salmonid fish species. It inhabits the waters of Sakhalin Island, the Kuril Islands, and rivers and lakes such as Sarufutsugawa River, Teshiogawa River, and Lake Shumarinai on Hokkaido Island, Japan. Sakhalin taimen lived once in Aomori and Iwate, Japan. They are eaten as sashimi, sushi, marinated, miso-grilled, in meunière sauce, deep-fried, *etc*. However, the non-edible portion, such as skin and bones, is produced during cooking. Most of it is currently discarded as waste, although the nutritional value of the skin and bones is high. Generally, these wastes account for approx. 20-80% of body mass, depending on the differences in the processing and the type of fish (*1*). Therefore, the non-edible portion needs to be reused in order to reduce the environmental impact, as it is a source of environmental pollution.

Collagen is a specific protein that is present in almost all tissues of animals, such as blood vessels, bones, cartilages, ligaments, skins and tendons. It is the most abundant protein in mammals, accounting for approx. 25-30% of total animal proteins (*2*). Because of bovine spongiform encephalopathy, foot-and-mouth disease, swine influenza, transmissible spongiform encephalopathy (*3*), or dietary restriction for religious reasons in the cultures of Hindus, Jews and Muslims, who make up 38.4% of global population (*4*), the acquisition of collagens from safer alternative sources is strongly desirable. Collagen, gelatine (partially hydrolysed collagen), and its peptides (collagen hydrolysates) (*5*) have been widely used not only in foods but also cosmetics, pharmaceutical and biomedical fields and in photography, due to excellent biocompatibility, high tensile strength and water holding property, and weaker antigenicity (*6*). At present, little information on the chemical properties of Sakhalin taimen has been reported, although it has been said that the Ainu people once ate the meat and used the skin for clothes and footwear in Hokkaido, Japan. The present study aims to isolate the collagen from Sakhalin taimen skin, prepare the succinylated collagen and elucidate its properties for industrial applications.

## MATERIALS AND METHODS

### Materials

Fresh Sakhalin taimen for the study was obtained from Ajigasawa Sakhalin taimen farm (Aomori, Japan). Collagen from bovine Achilles tendon and protein marker for SDS-PAGE were purchased from Nacalai Tesque Inc. (Kyoto, Japan). Lysyl endopeptidase from *Lysobacter enzymogenes* was obtained from Wako Pure Chemical Industries, Ltd. (Osaka, Japan). Toyopearl CM-650M was purchased from Tosoh Corp. (Tokyo, Japan). All chemicals were of reagent grade.

### Isolation of collagen from the skin

All the preparative procedures were carried out at 4 °C. The skins (crude lipid content on raw skin basis 6.5 g/100 g) were removed and cut into small pieces using a scalpel. To remove the non-collagenous proteins, samples were extracted with 20 volumes of 0.1 M NaOH for 2 days by changing the solution twice a day. They were filtered using the cheesecloth and then the residue was washed with distilled water until the pH value of the solution was adjusted as close as possible to neutral value. After filtration, the filtrate was treated with 5 volumes of cold acetone with gentle stirring for 2 days by changing the solution twice a day to remove the fats and pigments, followed by the treatment with 20 volumes of 0.5 M acetic acid under gentle stirring for 2 days. The obtained viscous solution was centrifuged at 50 000×*g* for 1 h at 4 °C using a refrigerated centrifuge (Himac SCR 20B; Hitachi-Koki Co., Ltd., Tokyo, Japan) with an angle rotor (RPR20-2; Hitachi-Koki Co., Ltd.). The supernatants were pooled, then NaCl was added to the solution at a final concentration of 0.9 M, followed by precipitation with 2.2 M NaCl in 0.05 M Tris-HCl buffer (pH=7.5) to purify the collagen. The samples were then centrifuged at 23 000×*g* for 30 min using the same refrigerated centrifuge with an angle rotor R14A (Hitachi-Koki Co., Ltd.), and the obtained precipitate was dissolved in a minimum volume of 0.5 M acetic acid. The samples were dialyzed against distilled water for 2 days by changing the solution twice a day and then lyophilized (crude lipid content on lyophilized collagen basis 0.05 g/100 g).

### Colour measurement

The colour of collagen is an important factor for industrial use. The colour of the lyophilized collagen was measured using a colourimeter (NR-11A; Nippon Denshoku Industries Co. Ltd., Tokyo, Japan) with illuminant D65 calibrated to black and white standards. The results were shown as the mean value±standard deviation of ten measurements.

### Sodium dodecyl sulfate-polyacrylamide gel electrophoresis and peptide mapping

To determine the purity of the obtained collagen and compare the patterns of peptide fragments on Sakhalin taimen skin collagen with bovine Achilles tendon collagen, SDS-polyacrylamide gel electrophoresis (SDS-PAGE) (7.5% gel) and peptide mapping (10% gel) were performed as described previously (*7*). For the peptide mapping, the collagen samples (0.5 mg) were dissolved in 0.1 M sodium phosphate buffer (pH=7.2) containing 0.5% SDS and heated at 100 °C for 5 min to allow the effective enzyme digestion. After cooling in ice, the denatured collagen samples were digested at 37 °C for 30 min using lysyl endopeptidase (0.24 amidase activity). After the addition of SDS to a final mass fraction of 2%, the samples were boiled for 5 min and then used for SDS-PAGE.

### Ultraviolet absorption spectrum

Type I collagen shows a distinct ultraviolet absorption spectrum. To determine the composition of collagen the ultraviolet absorption spectrum was analysed at 220-350 nm using a UV/Vis spectrophotometer (V-530; JASCO Co., Tokyo, Japan) as described previously (*8*).

### Subunit composition

The subunit composition of collagen was confirmed by column chromatography and SDS-PAGE. The subunit components of the obtained collagen sample were separated using a Toyopearl CM-650M column (1.0 cm×5.0 cm) as described previously (*7*). The absorbance of the components was measured at 230 nm using a UV/Vis spectrophotometer, and then the components were analysed by SDS-PAGE using 7.5% polyacrylamide gel.

### Amino acid composition

Collagen was hydrolyzed in 6 M HCl for 24 h at 110 °C under reduced pressure (approx. 2 kPa), and then the amino acid composition of the hydrolysates was analysed on a JASCO liquid chromatography system (LC-2000*Plus*; JASCO Co., Tokyo, Japan) using online precolumn derivatization with *o*-phthalaldehyde. The excitation and emission wavelengths were set at 345 and 455 nm, respectively. Simultaneously, the analysis was performed using amino acid standard solution.

### Denaturation temperature

Denaturation temperature of the collagen was measured as temperature that caused 50% decrease in viscosity as described previously (*7*). The viscosity of the collagen was determined using a Cannon-Fenske type viscometer with an average shear gradient of 400 s^-1^ (SIBATA Scientific Technology Ltd., Saitama, Japan). Each point was expressed as the mean value±standard deviation of six determinations. The fractional viscosity was calculated as follows:

(*η*_sp_/*c*)*_t_*/(*η*_sp_/*c*)*_t_*_=10 °C_ /1/

where *η*_sp_/*c* is reduced viscosity (in mL/g), *η*_sp_ is specific viscosity of collagen and *c* is the concentration of the solution (in g/mL).

### Attenuated total reflectance-Fourier transform infrared spectroscopy

Attenuated total reflectance-Fourier transform infrared (ATR-FTIR) spectra were measured at 20 °C and 40% relative humidity by coupling ATR accessory (ATR PRO410-S; JASCO Co., Tokyo, Japan) to a JASCO FT/IR-4100 spectrometer (JASCO Co., Tokyo, Japan). The spectra were obtained over the range of 4000-650 cm^-1^ at 4 cm^-1^ resolution. In addition, the resultant spectra were analysed to confirm the composition of the secondary structural components of collagens using a FTIR Protein Secondary Structure Analysis Program (JASCO Co.).

### Succinylation of collagen

The succinylated collagen was prepared at 4 °C. Collagen was dissolved in 0.5 M acetic acid, and then the pH value of the solution was adjusted to 10 using 5 M NaOH. An equal mass of succinic anhydride t was added slowly to the solution. The pH value of the solution was maintained in the range of 9-10 by adding NaOH. After gentle stirring for a day, the pH value of the solution was adjusted to 4.2 with 4 M HCl, and then the solution was centrifuged (Himac SCR 20B; Hitachi-Koki Co., Ltd.) at 50 000×*g* for 30 min. The obtained precipitate was dissolved in a minimum volume of 0.5 M acetic acid, dialyzed against distilled water and then lyophilized to obtain the succinylated collagen.

### Viscosity of succinylated collagen solution

The viscosities of the untreated and succinylated collagen solutions (0.1% *m*/*V*) were determined at 20 °C using a viscometer (TVC-7; Toki Sangyo Co., Ltd., Tokyo, Japan). The solvents used to prepare the solution for the measurements were as follows: 0.1 M acetic acid for the untreated collagen and distilled water (pH=6.0) for the succinylated collagen.

### Solubility of succinylated collagen

It is necessary to solubilize the collagen under the physiological conditions for industrial applications. The succinylated collagen (1 mg) was suspended in 10 mL distilled water at different pH values and was then gently stirred for a day at 4 °C. The samples were centrifuged at 50 000×*g* and 4 °C for 1 h, and the supernatants were used for determination of the protein content (*9*).

### Statistical analysis

Except for colour and denaturation temperature measurements, each assay was repeated three times independently. The results were reported as mean value±standard deviation. Significant differences were tested by one-way analysis of variance with the Tukey’s range test (p<0.05). Minitab Statistical software (*10*) was used for statistical analyses.

## RESULTS AND DISCUSSION

### Collagen yield

Collagen was solubilized with acetic acid, and then odourless and pure white (L*=95.10±1.07, a*=-0.04±0.02, b*=1.32±0.13) collagen was obtained with high yield of approx. (38.3±3.5) % on dry skin mass basis (approx. 11.4% on raw skin mass basis). In our previous reports, collagens were prepared from the skins of the aquatic organisms with high yields of approx. 21.4-51.4% on dry mass basis (*11*-*16*). Kittiphattanabawon *et al*. (*17*) and Savedboworn *et al*. (*18*) reported that collagens could be obtained from the skin of the fish from Thailand with high yields of approx. 27.6-64.2%. Recently, ultrasound extraction (*19*) and sonication (*20*) have been developed for the extraction of collagen. These methods may increase the efficiency and reduce the time and cost of the extraction of collagen compared to the conventional extraction methods.

### Determination of molecular mass of Sakhalin taimen skin collagen

**Fig. 1:**
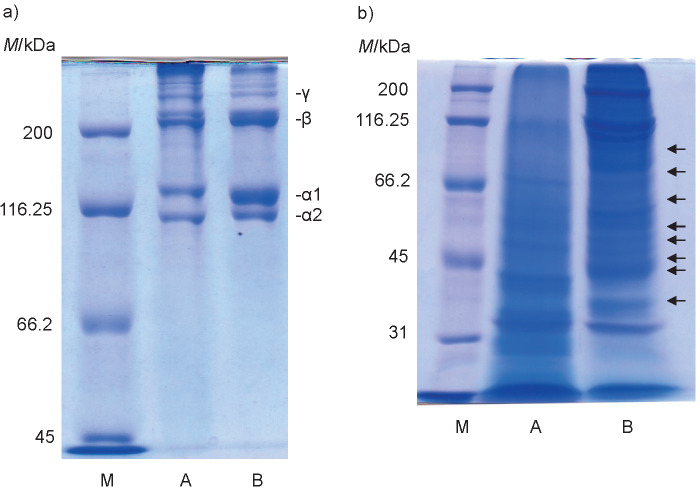
Results of: a) SDS-polyacrylamide gel electrophoresis analysis, and b) peptide mapping of the collagens. M=molecular marker proteins, A=bovine Achilles tendon collagen, B=Sakhalin taimen skin collagen

As shown in Fig. 1, two distinct α chain bands, α1 and α2, were detected with the molecular mass of 140 and 130 kDa, respectively. A large number of the β chains with the molecular mass of 220 kDa was observed. The existence of the α3 chain was not confirmed under these conditions. The results suggested that Sakhalin taimen skin collagen was type I collagen with a chain composition of (α1)_2_α2 heterotrimer or α1α2α3 heterotrimer. Simultaneously, bovine Achilles tendon collagen was analysed by SDS-PAGE under the same conditions. The molecular mass (140 kDa) of the α1 chain was similar to that of Sakhalin taimen skin collagen, however, the molecular mass (120 kDa) of the α2 chain was slightly smaller than that of Sakhalin taimen skin collagen. Therefore, the molecular mass (200 kDa) of the β chains as cross-linked α chains was smaller than that of Sakhalin taimen skin collagen. In contrast, the collagen from sole fish skin consisted of two α chains, with the molecular mass of 118 (α1) and 116 kDa (α2), respectively, and of the β chain with the molecular mass of 200 kDa (*2*). Cheng *et al*. (*21*) reported that collagen from jellyfish *Rhopilema esculentum* mesogloea showed the electrophoretic patterns with high molecular mass of α2 chain in comparison with that of α1 chain. Matsui *et al*. (*22*) investigated the subunit composition of type I collagens from fish species in Salmonidae family. The skin collagens were of type I with a chain composition of α1α2α3 heterotrimer in fish species such as chum salmon, coho salmon, Japanese char, masu salmon and rainbow trout. In contrast, in all fish species belonging to Osmeridae, Plecoglossidae, and Salangidae families, the chain composition of the skin collagens was (α1)_2_α2. These results suggest that since Sakhalin taimen belongs to Salmonidae family, its skin collagen may consist of chains of α1α2α3 heterotrimers.

### Comparison of collagen cleavage sites for lysyl endopeptidase

The peptide mapping was performed to easily compare the primary structure of Sakhalin taimen skin collagen with that of bovine Achilles tendon collagen. It was determined that the cleavage sites of Sakhalin taimen skin collagen for lysyl endopeptidase were fairly different from those of bovine Achilles tendon collagen (Fig. 1), indicating that primary structure of these two collagens is considerably different. The skin collagens of fish species belonging to Salmonidae family were digested using V8 protease from *Staphylococcus aureus*, and then the peptide mapping was performed (*22*). These cleavage sites were similar among these fish species. Thus, it is suggested that the primary structures of skin collagens of fish species from the same family are similar.

### Ultraviolet absorption spectrum of Sakhalin taimen skin collagen

The maximum and minimum peaks on Sakhalin taimen skin collagen were shown at 235 and 222 nm, respectively (data not shown). The absorption was not detected at 280 nm, suggesting the absence of tryptophan residue in the collagen (Table 1). In addition, the absorption between 250 and 290 nm (data not shown) was not observed, suggesting low content of phenylalanine and tyrosine (Table 1). The absorption peak around 230 nm is attributed to the peptide bond absorption by n→π* transitions of the groups of C=O, -COOH and CONH_2_ in the polypeptide chains (*23*). The maximum absorption of type I collagen from marine organisms was reported as follows: bluefin tuna skin 238 nm (*24*), channel catfish skin 232 nm (*25*), largefin longbarbel catfish skin 233 nm (*26*) and red drum fish scales 230 nm (*27*).

**Table 1:** Amino acid composition of collagen from Sakhalin taimen skin (amino acid residues per 1000 total amino acid residues)

Amino acid	Residue
Hydroxyproline	77
Hydroxylysine	6
Aspartic acid	44
Threonine	25
Serine	37
Glutamic acid	71
Proline	115
Glycine	352
Alanine	118
Valine	16
Methionine	12
Isoleucine	10
Leucine	20
Tyrosine	2
Phenylalanine	13
Lysine	25
Histidine	5
Arginine	52
Total	1000
DH/% (proline)	40.1
DH/% (lysine)	19.4

DH=degree of hydrolysis

### Subunit composition of Sakhalin taimen skin collagen

**Fig. 2:**
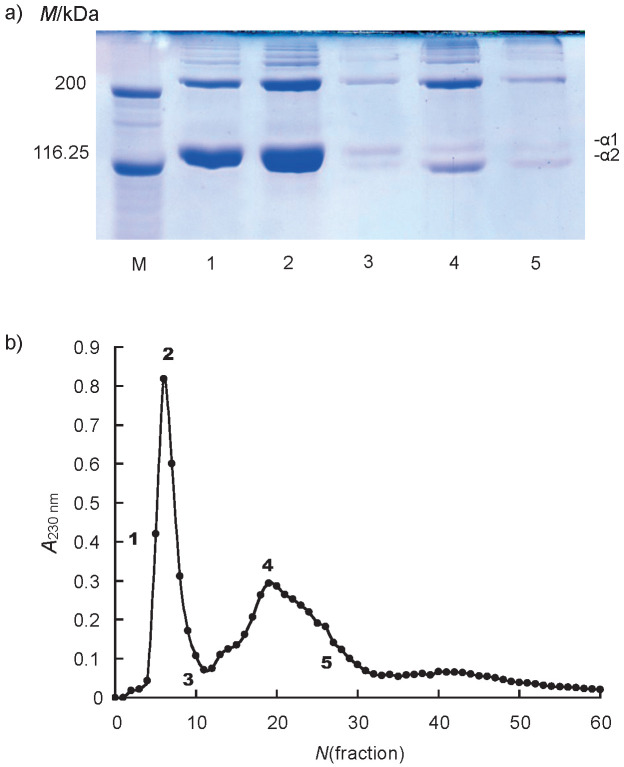
Results of: a) SDS-polyacrylamide gel electrophoresis analysis of the fractions indicated by numbers, and b) Toyopearl CM-650M column chromatography of the denatured Sakhalin taimen skin collagen

The α chains of Sakhalin taimen skin collagen were separated to two major protein fractions (Fig. 2). The α1 chain was detected in fractions 1 and 2, and the α2 chain was detected in fractions 4 and 5. However, the α3 chain was not detected under these conditions (Fig. 2). Thus, Sakhalin taimen skin collagen was a heterotrimer with a chain composition of (α1)_2_α2. Kittiphattanabawon *et al*. (*17*) reported that clown featherback skin collagen was (α1)_2_α2 heterotrimer. In contrast, brown backed toadfish skin collagen was α1α2α3 heterotrimer (*28*). The α3 chain exists in many teleost skin collagens, although it was not detected in cyclostome and cartilaginous fish skin collagens, which lack type I collagen α3 gene. In fact, our group revealed that the α3 chain existed in skin collagens of aquatic organisms including surf smelt (*11*-*13*, *16*). In addition, Matsui *et al*. (*22*) reported that the α3 chain existed in the skin collagens of all salmonid fish, such as cherry salmon, chum salmon, coho salmon, Japanese char, rainbow trout, *etc*. In contrast, the subunit composition of the skin collagens was (α1)_2_α2 heterotrimer in capelin and Japanese smelt belonging to Osmeridae, ayu belonging to Plecoglossidae, and icefish belonging to Salangidae family among fish species in the suborder Salmonoidei. These results suggested that the α3 chain was not detected in teleost skin collagens including Sakhalin taimen because of a very low or lack of the expression of α3 gene. Thus, this finding was the first report on the absence of the α3 chain in salmonid fish skin collagens. The differences in the functional properties of collagens with or without α3 chain have not been investigated in any detail.

### Amino acid composition of Sakhalin taimen skin collagen

Glycine was the most abundant amino acid in Sakhalin taimen skin collagen (Table 1). The contents of the following amino acids were relatively high: alanine (118 residues), proline (115 residues), hydroxyproline (77 residues) and glutamic acid (71 residues). In contrast, the contents of tyrosine, histidine, hydroxylysine, isoleucine, methionine, and phenylalanine were low, and cysteine and tryptophan were not detected at all. These are all typical amino acids for type I collagen, which contains a large amount of hydroxyproline and a small amount of hydroxylysine.

Next, the imino acid (proline and hydroxyproline) content in Sakhalin taimen skin collagen was calculated to be 192 residues (Table 1). It was higher than those of skin collagens from brown backed toadfish (*28*), channel catfish (*25*), deep-sea redfish (*29*), and ocellate puffer (*14*), however, it was lower than those of skin collagens from brownstripe red snapper (*30*), clown featherback (*17*) and largefin longbarbel catfish (*26*). In contrast, the content was similar to those of skin collagens from cuttlefish (*11*, *15*), golden pompano (*31*), grass carp (*32*) and walleye pollack (*33*). In general, the collagens with higher imino acid content have greater helix stability.

The hydroxylation degree of the proline residues of Sakhalin taimen skin collagen was also calculated. The degree was 40.1% (Table 1), which was similar to those of skin collagens from bigeye snapper (*34*) and Nile perch (*35*). On the other hand, it was higher than those of skin collagens from brownstripe red snapper (*30*), golden pompano (*31*), grass carp (*32*), largefin longbarbel catfish (*26*) and ocellate puffer (*14*). However, it was lower than those of skin collagens from brown backed toadfish (*28*), channel catfish (*25*), clown featherback (*17*) and cuttlefish (*11*, *15*). The thermal stabilities of the collagens are different among fish species. In addition, the hydroxylation degree of proline has direct effect on the thermal stability of the collagen. That is, the stability is proportional to the hydroxyproline content of the collagen. It is suggested that fish species like poikilotherms slightly control the hydroxylation degree of the proline residues in the collagen, regulate the hydroxyproline content and provide the thermal stability to type I collagen depending on the body temperature.

### Denaturation temperature of Sakhalin taimen skin collagen

**Fig. 3:**
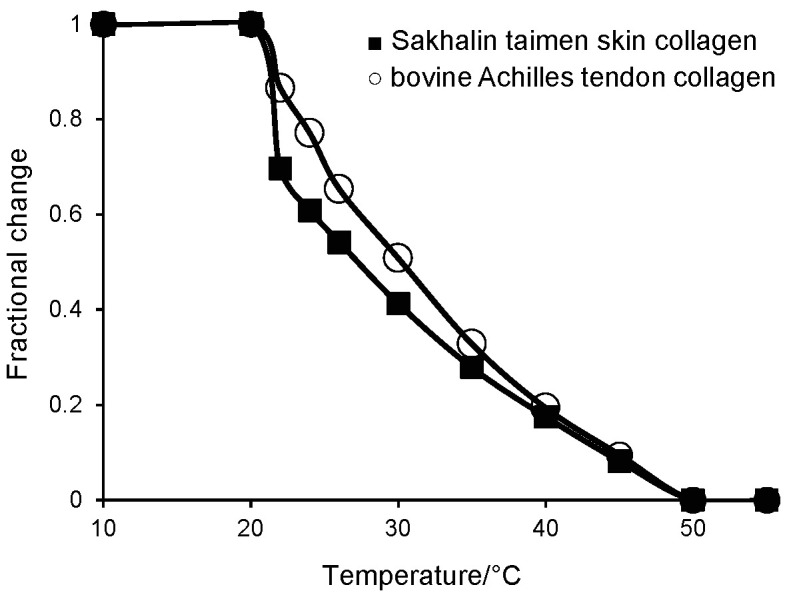
Thermal denaturation curves of the collagens from Sakhalin taimen skin and bovine Achilles tendon. The denaturation temperatures were measured as the 50% decrease of viscosity in 0.1 M acetic acid. The incubation time at each temperature was 30 min

The denaturation temperature is a temperature at which the helical structures of the collagen molecules break down in the solution, and then change to gelatin with random structures. The denaturation temperature of Sakhalin taimen skin collagen was calculated at approx. 27.3 °C, which was 4 °C lower than that of bovine Achilles tendon collagen (Fig. 3). It was similar to that of skin collagens from brown backed toadfish (*28*), carp (*36*), cuttlefish (*11*, *15*), grass carp (*32*), jellyfish (*37*, *38*), ocellate puffer (*14*), octopus (*13*) and walleye pollack (*33*). It is suggested that the denaturation temperature of collagen from aquatic organisms is lower than that of the collagen from terrestrial animals. Denaturation temperature of collagen from warm-water species, such as bigeye snapper (*34*), channel catfish (*25*) and largefin longbarbel catfish (*26*), is lower than that of cold-water species, such as deep-sea redfish (*29*). Kimura (*39*) investigated the denaturation temperature and hydroxylation degree of the proline residues in ordinary muscle and skin collagens of some fish species, such as carp, chub mackerel, chum salmon, eel, Pacific saury and skipjack tuna. Denaturation temperature of ordinary muscle collagen is approx. 1 °C higher than that of skin collagen in all fish species. Moreover, the hydroxylation degrees of the proline residues in ordinary muscle collagens are higher than those of skin collagens. That is, this reflects the fact that the temperature of the inside of the fish body is slightly higher than that on the surface of the body. Thus, the denaturation temperature of collagen is related to the environmental temperature and body temperature. In addition, it is known that the hydroxyproline content of the collagens is positively correlated with the denaturation temperature of the collagens.

### ATR-FTIR spectroscopy analysis

**Fig. 4:**
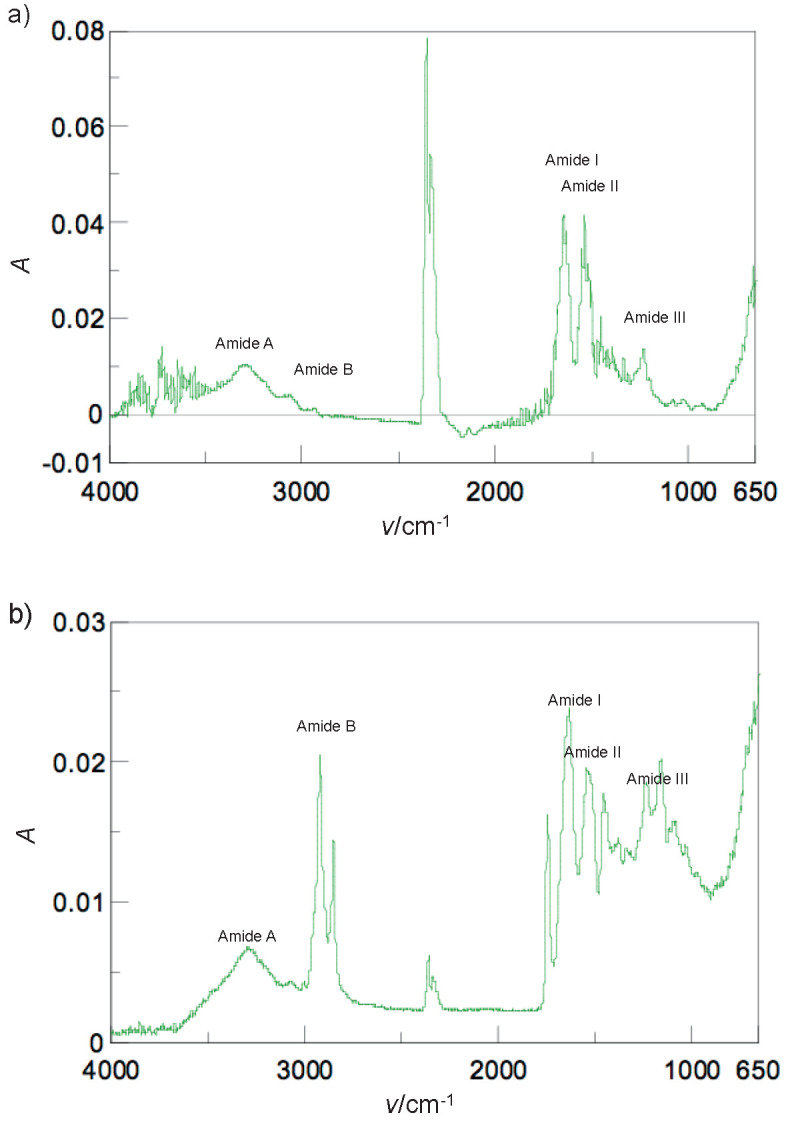
Attenuated total reflectance-Fourier transform infrared spectra of the collagens from: a) Sakhalin taimen skin, and b) bovine Achilles tendon

The ATR-FTIR spectrum of Sakhalin taimen skin collagen is shown in Fig. 4. The free NH-stretching vibration occurs at 3400 to 3200 cm^-1^. The amide A band is related to the NH-stretching frequency. Its position is shifted to low frequencies (3300 cm^-1^), as the NH-group of the peptide is coupled by strong hydrogen bond among the molecules. The amide A band of Sakhalin taimen skin collagen was observed at 3310.21 cm^-1^, indicating the existence of hydrogen bonds in the collagen molecule. The amide B band (around 3100 cm^-1^) is related to the NH-stretching and the overtone of amide II as a result of Fermi resonance. The amide B band, which was found at 2946.70 cm^-1^, is associated with the CH_2_-asymmetrical stretch. The amide I and II bands are sensitive markers of the peptide secondary structure. The amide I band occurs at around 1650 cm^-1^ (from 1700 to 1600 cm^-1^) and is related to the stretching vibrations of C=O bond. The amide I band was shown at 1645.95 cm^-1^, indicating the C=O stretching vibration or the existence of the hydrogen bond coupled with COO^-^. The amide II band occurs at 1650 to 1500 cm^-1^, as a consequence of the NH-bending vibration coupled with the CN-stretching. Collagen from Sakhalin taimen skin showed the amide II band at 1539.88 cm^-1^. Moreover, the amide III band is observed at 1320 to 1200 cm^-1^ and is associated with the NH-bending vibration coupled with the CN-stretching. The amide III band was observed at 1234.22 cm^-1^. These results indicated the existence of the helical arrangements in Sakhalin taimen skin collagen. In addition, strong CH-stretching vibration in Sakhalin taimen skin collagen was observed at 2361.41 cm^-1^. Generally, this occurs between 2854 and 1745 cm^-1^. Moreover, the bands of bovine Achilles tendon collagen were detected as follows: amide A (3294.79 cm^-1^), amide B (2926.45 cm^-1^), amide I (1633.41 cm^-1^), amide II (1542.77 cm^-1^) and amide III (1238.08 cm^-1^) (Fig. 4).

Next, the percentage of the secondary structural components in Sakhalin taimen skin collagen was calculated. The results were as follows: 23% α-helix, 27% β-sheet, 22% β-turn and 24% others, such as random coil structure. In bovine Achilles tendon and common minke whale *unesu* collagens they were 9, 35, 20 and 22% and 15, 45, 16 and 18%, respectively (*7*). Sakhalin taimen skin collagen had richer α-helix and poorer β-sheet structures than the collagen of this mammal. Thus, it was concluded that the secondary structure of Sakhalin taimen skin collagen greatly differs from that of mammals.

### Properties of succinylated collagen

In general, collagen is dissolved in acidic pH, such as in diluted acetic, citric and hydrochloric acids. However, collagen dissolved in acids cannot be used in many applications. Chemical modification is a useful technique for the improvement of the functional properties of proteins. Among them, succinylation occurs in the reaction of ε-amino group in lysine residues and N-terminal α-amino group of proteins after the addition of succinic anhydride. It has been used in the modification of the physiological properties, such as the structure and thermal aggregation, of soy protein isolate, β-conglycinin and glycinin (*40*). As a result, it can improve the stability of soy proteins after heating.

**Fig. 5:**
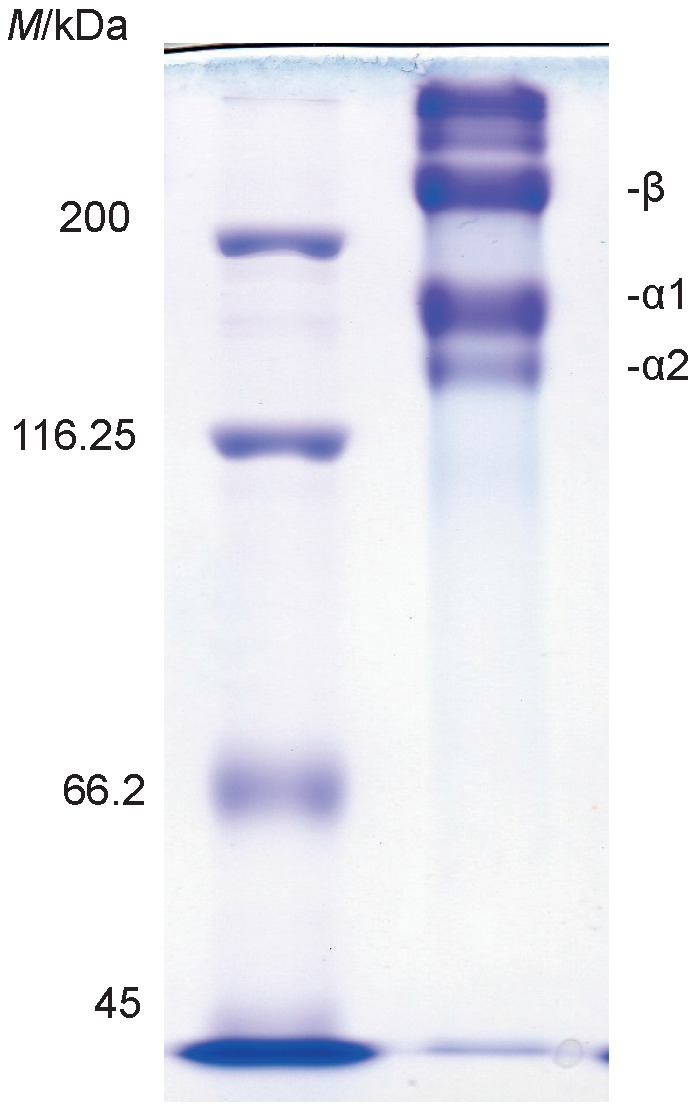
Results of SDS-polyacrylamide gel electrophoresis analysis of the succinylated Sakhalin taimen skin collagen. Left lane=molecular marker proteins, right lane=succinylated collagen

The succinylated collagen from Sakhalin taimen skin was prepared. As shown in Fig. 5, two distinct α chain (α1 and α2) and one β chain bands were detected as well as those of the untreated collagen (Fig. 1). The molecular masses of these bands were approx. 170, 145 and 245 kDa, respectively. Zhang *et al*. (*41*) reported that the succinylated pepsin-solubilized collagen from calfskins had fewer migrated subunits than its pepsin-solubilized collagen in SDS-PAGE patterns, suggesting the increase of its molecular mass by introduction of succinyl residues. Thus, succinylation of the collagen could be easily checked by SDS-PAGE analysis.

**Fig. 6:**
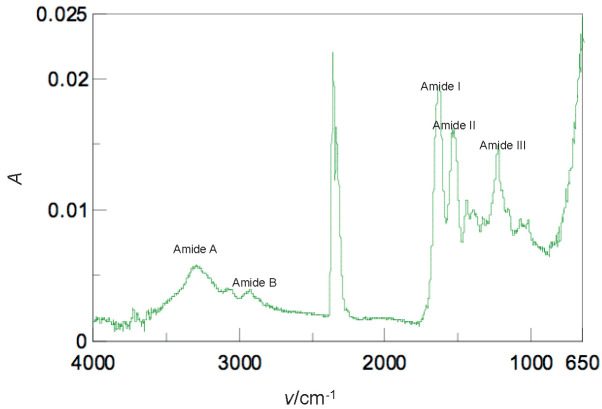
Attenuated total reflectance-Fourier transform infrared spectrum of the succinylated Sakhalin taimen skin collagen

ATR-FTIR spectrum of the succinylated collagen from Sakhalin taimen skin is shown in Fig. 6. The bands of the succinylated collagen were observed as follows: amide A (3295.75 cm^-1^), amide B (2931.27 cm^-1^), amide I (1644.98 cm^-1^), amide II (1538.92 cm^-1^) and amide III (1237.11 cm^-1^). The positions of the succinylated collagen were not shifted in comparison with those of the untreated collagen. The rate of the secondary structural components in the succinylated collagen was as follows: 13% α-helix, 32% β-sheet, 19% β-turn and 21% other structures. Thus, succinylation caused the secondary structural changes (the decrease of the α-helix content and the increase of the β-sheet content) of the collagen molecule. Wan *et al*. (*40*) investigated the secondary structures of the untreated and succinylated soy protein isolates using a far-UV circular dichroism spectropolarimeter. The β-sheet content of β-conglycinin decreased with increasing succinylation degrees, although the α-helix content was stable. In contrast, the α-helix content of glycinin decreased, whereas the β-sheet content gradually increased. Succinylation lead to the destruction of the secondary structures of the proteins, such as β-lactoglobulin and soy protein hydrolysates (*42*). However, it was reported that succinylation made little impact on the secondary structures of bovine serum protein and lysozyme (*43*).

The derivation temperature of the succinylated collagen from Sakhalin taimen skins was estimated at approx. 27.5 °C. It was similar to that of the untreated collagen from Sakhalin taimen skin. In contrast, the derivation temperature (34.7 °C) of succinylated pepsin-solubilized collagen from calfskin was 4 °C lower than that (38.4 °C) of pepsin-solubilized collagen (*41*), although these collagens were dissolved in different solvents.

The viscosities of the untreated and succinylated collagen solution from Sakhalin taimen skin were measured. The viscosity of the untreated collagen solution was estimated to (35.2±0.2) mPa∙s (data not shown). On the other hand, the succinylated collagen solution showed approx. 21-fold higher viscosity ((726.3±0.4) mPa∙s) than the untreated collagen solution. Thus, it could be concluded that succinylation increased the viscosity.

**Fig. 7:**
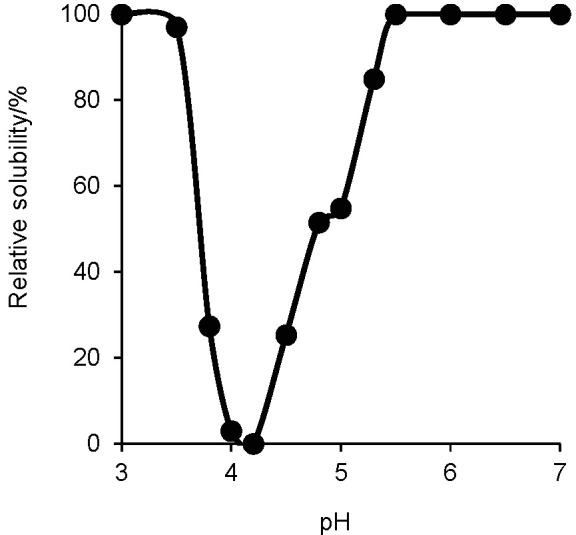
Relative solubility of the succinylated Sakhalin taimen skin collagen obtained by dividing protein content measured at different pH values with protein content at pH=3

The solubility of the succinylated collagen from Sakhalin taimen skin was investigated under different pH conditions. It completely solubilized in the ranges from pH=3.0-3.5 to 5.5-7.0 (Fig. 7). In contrast, it hardly solubilized at pH=4.2.

Collagen is most abundant protein in the bodies of fish and animals, and is used for various applications as a biomaterial due to its excellent characteristics. For example, collagen can be used in the production of edible sausage casings and gelatin in food industry, and as a haemostatic agent, for repair of sunken recess in the skin, vitreous implants and wound dressings in health care. Collagen can be solubilized at physiological pH using a chemical modification technique such as succinylation. Therefore, there is a possibility that the application of collagen will expand in other fields even more. In contrast, the lack of α3 chain in Sakhalin taimen skin collagen is critically interesting in comparative biochemical studies of skin collagens in Salmonidae family. We aim to elucidate the physicochemical and functional properties of Sakhalin taimen skin collagen in the near future.

## CONCLUSIONS

We tried to isolate the collagen from Sakhalin taimen skin, prepare the succinylated collagen and elucidate its properties for industrial applications. In summary, cold acetone treatment was an effective method for the removal of the fats and pigments from Sakhalin taimen skin. A high yield of odourless and pure white collagen was obtained. Succinylation increased the viscosity and improved the solubility of Sakhalin taimen skin collagen under the physiological conditions at around pH=6. Collagen from Sakhalin taimen skin can be effectively utilized as an alternative to terrestrial animal collagen, not only in food industry but also in cosmetics, pharmaceuticals, biomaterials and biomedicals.
